# Clinical Benefit of Liver Stiffness Measurement at 3 Months after Kasai Hepatoportoenterostomy to Predict the Liver Related Events in Biliary Atresia

**DOI:** 10.1371/journal.pone.0080652

**Published:** 2013-11-18

**Authors:** Seung Min Hahn, Seung Kim, Kook In Park, Seok Joo Han, Hong Koh

**Affiliations:** 1 Department of Pediatrics, Yonsei University College of Medicine, Severance Pediatric Liver Disease Research Group, Severance Children's Hospital, Seoul, Korea; 2 Department of Pediatric surgery, Yonsei University College of Medicine, Severance Pediatric Liver Disease Research Group, Severance Children's Hospital, Seoul, Korea; The University of Hong Kong, Hong Kong

## Abstract

**Background:**

The progression of hepatic fibrosis may result in decompensated hepatic failure with cirrhosis, liver related events (LRE) such as ascites, variceal bleeding, and death after successful and timely Kasai hepatoportoenterostomy (HPE) in biliary atresia. The aim of this study is to suggest clinical benefit of the liver stiffness measurement (LSM) using transient elastography at 3 months after the Kasai operation to predict LRE.

**Methods:**

Between January 2007 and December 2011, 69 eligible biliary atresia patients who underwent Kasai HPE and performed transient elastography before and 3 months after HPE were included. The occurrences of LRE were analyzed for all patients. All patients were divided into 2 groups (with and without LRE) for comparison. Multivariate analysis was used to detect the independent risk factors of LRE. The area under the receiver operation characteristics curve (AUROC) was used to establish the LSM optimal cutoff value of 3 months after Kasai operation in predicting LRE.

**Results:**

LSM value, aminotransferase, albumin, bilirubin, and PT-INR significantly differed among the two groups. Multivariate analysis demonstrated LSM value as the most powerful independent factor of the development of LRE. The cut-off value of 19.9 kPa was calculated to be optimal for predicting LRE development with total sensitivity and specificity of 1.804. AUROC resulted in 0.943, with sensitivity of 85.3% and specificity of 95.2%.

**Conclusions:**

The LSM value of 3 months after Kasai HPE can be a useful predictor of LRE development.

## Introduction

Since the introduction of hepatoportoenterostomy (HPE) by Dr. Kasai in the 1950s, it has been the treatment of choice for biliary atresia patients. More than 90% of patients undergo Kasai HPE after diagnosis of biliary atresia while some patients with progressed end stage liver disease chose the transplantation first, although liver transplantation is performed during follow-up on up to 50% of children who already had the Kasai operation. [1] The most common reason for secondary liver transplantation is failure of the Kasai operation, which presents as persistent cholestasis and progression of liver cirrhosis. [2] However, even if a patient has underwent a successful and timely operation, this progression of histological fibrosis occurs in almost all patients, [3] leading to biliary atresia as the main indication of children's liver transplantation. Therefore, long-term follow-up of biliary atresia patients with close monitoring of fibrosis and cholestasis is important.

Progression of subclinical compensated liver disease may result in decompensated hepatic failure with cirrhosis, liver related events (LRE) such as ascites, variceal bleeding, hepatic encephalopathy and death. Although clinical features and laboratory data can be helpful, definitive diagnosis of hepatic fibrosis and cirrhosis is classically performed with a histological examination of liver tissue obtained from a liver biopsy. Due to the limitations (such as invasiveness and excessive pain) involved in performing liver biopsy, alternative methods for diagnosis of hepatic fibrosis have been emerging. [4]

The hepatic fibrosis scan (Fibroscan, Echosens, Paris, France), a new medical device, allows for a noninvasive, rapid, painless, and reproducible method, [5], [6] is based on transient elastography to measure liver stiffness. Good reproducibility with low variability has been reported in many previous studies. [5], [7], [8] Analyses using the intra-class correlation coefficient and correlation with different patient-related and liver disease–related covariates have previously shown intra- and inter-observer agreements. The overall inter-observer agreement and intra-class correlation coefficient was 0.98 (95% confidence interval [CI] 0.977–0.987). [7]

The results measured by Fibroscan correlates well with the histological stage of liver fibrosis. [8] Liver volume measured by Fibroscan is at least 100 times larger than usual liver biopsy samples; therefore, results from Fibroscan are expected to be a more representative sample of the whole hepatic parenchyma. [7]

Since the first report using the Fibroscan in 2003, [5], [9] it has been used and validated in many hepatic diseases. In addition, its feasibility in children as well as in adults has been identified. [10] Previous studies have discussed the relationship between liver stiffness measured by Fibroscan and the liver associated complications in adults with chronic liver disease; liver stiffness measurement (LSM) may be useful as a predictor of the development of hepatocellular carcinoma and hepatic decompensating events in chronic hepatitis B. [11], [12]

LSM has proved it's feasibility in children even younger than 24 months [13] with various liver diseases. [14] Some studies on children compared liver LSM after biliary atresia between patients with and without esophageal/gastric varices. [15], [16] However, few studies on children have focused on overall complications of liver failure using LSM. Therefore, the aim of this study is to determine a clinical benefit of LSM at 3 months after the Kasai operation in order to predict LRE.

## Materials and Methods

### Ethics Approval

This study was approved by the ethical committee from Yonsei University College of Medicine, Severance Hospital, and study protocol was in accordance with the Declaration of Helsinki. Data was collected from the medical records and stored in a locked study member's computer without patients' identifiers. The consent was not obtained, because it was a non-invasive study and specifically waived by the approving institutional review board (IRB).

### Patients

Between January 2007 and December 2011, a total of 69 children who underwent Kasai HPE at a single center and had Fibroscan examination before and 3 months after a Kasai operation were included in the study. In addition, the occurrence of LRE was analyzed for 69 children.

Exclusion criteria were as follows: 1) LSM failure (no valid shots), 2) invalid LSM [defined as an interquartile range (IQR) to median value ratio (IQR/M)>0.3], success rate <60%, or <10 valid measurement], 3) a history of hepatic decompensation, 4) co-infection with hepatitis B, hepatitis C, hepatitis D or HIV, 5) right-sided heart failure and 6) loss to follow-up. No patients were excluded.

A total of 69 children were divided into two groups: one group who had developed LRE and one group without any event after Kasai HPE. A comparison between the groups was performed. Among several factors, the independent risk factors of LRE development were accessed using multivariate analysis. The areas under the receiver operating characteristics curve (AUROC) were used to establish the LSM cutoff value of 3 months after the Kasai operation in predicting LRE.

### Laboratory Tests & Liver Histopathology

Laboratory examinations were collected 3 times: on the same day of the Kasai operation (baseline study), 3 months after the surgery, and during follow-up. Data including aspartate amino-transferase (AST), alanine amino-transferase (ALT), serum albumin, serum bilirubin, gamma-glutamyl transpeptidase (GGT), alkaline phosphatase (ALP), prothrombin time (PT-INR), and platelet count were recorded. The upper normal range of AST and ALT were 40 IU/L.

Liver specimens were obtained during Kasai operation and were examined by an experienced pathologist. The METAVIR scoring system, [17] which consists of 5 stages, based on the architectural features of portal fibrosis, was used. (F0 =  no fibrosis, F1 =  portal fibrosis without septa, F2 =  portal fibrosis and few septa, F3 =  numerous septa without cirrhosis, and F4 =  cirrhosis).

### LSM

Liver stiffness was measured before the Kasai operation and at a follow-up period of 3 months after the Kasai procedure using Fibroscan. The methods were similar to those of our previous study. [16] The right lobe of the liver through the intercostal spaces was targeted until 10 successful measurements were obtained. Each child was placed in the supine position with maximal abduction of their right arm. The median value among 10 measurements of liver stiffness was calculated automatically by software on a microcomputer installed in the Fibroscan, and is expressed in kilopascals (kPa). A success rate of LSM of at least 60% has been considered reliable. [16], [18]

### Definition of LRE

LRE in this study included esophageal/gastric variceal bleeding, ascites, prolonged jaundice, hepatic encephalopathy, hepato-renal syndrome and death. Variceal bleeding was diagnosed by endoscopic evaluation when bleeding developed from the enlarged veins in the distal esophagus or gastric fundus. [19] An imaging study such as ultrasonography or computed tomography was used to diagnose ascites; fluid collection in the abdomen related with cirrhosis was observed in the imaging study. [20] Jaundice (serum bilirubin ≥5 mg/dl [85//µmol/l]) was defined according to the guidelines of Asian Pacific Association for the study of the liver. [21] Liver related death was defined as death caused by the complications of end-stage liver disease during follow-up of biliary atresia after Kasai operation.

### Statistical Analysis

Data was written as mean ± standard deviation (SD), median value with range, and N (%). The baseline characteristics of subjects were compared using the chi-squared and Fisher's exact tests. To document the independent effective factors of LRE development, univariate and multivariate Cox proportional hazard regression analyses were performed. Corresponding 95% confidence intervals and hazard ratio (HR) are demonstrated in the study. In addition, AUROC values, which maximized the sum of sensitivity and specificity, were described to identify the optimal cutoff value of LSM predicting LRE development. The cumulative incidence rates of LRE were calculated using the Kaplan-Meier method with log-rank test. All statistic analyses were performed using the SPSS statistical software (version 18.0, SPSS Inc., Chicago, IL). A P value <0.05 was considered to be statistically significant.

## Results

### Characteristics of All Patients

A total of 69 patients (19 male and 50 female) were studied. The characteristics of each child at baseline and at 3 months after the operation are shown in **Table 1**. The mean age before operation was 10.66±6.03 weeks, and the AST and direct bilirubin were 146.5±108.9 and 7.04±2.75, respectively. The median LSM value was 10.3 kPa before surgery and 16.5 kPa after 3 months in all patients. F3 and F4 METAVIR fibrosis stages were noted in 8 (11.6%) and 10 (14.5%) patients.

**Table 1:** Characteristics of baseline and 3 months after HPE in all patients.

Characteristics	All patients (N = 69)	
	Baseline	3 month after
Male (%)	19(27.5)	
Age (weeks)	10.66±6.03	
METAVIR fibrosis stage, 3/4	8/10	
LSM value (kPa)	10.3 (3.1–45.0)	16.5 (3.5–75.0)
AST (IU/L)	146.5±108.9	173.6±142.5
ALT (IU/L)	126.4±94.7	164.7±102.1
Albumin (g/dL)	3.81±0.44	3.48±0.71
Total bilirubin (mg/dL)	8.94±3.02	4.79±5.24
Direct bilirubin (mg/dL)	7.04±2.75	4.05±4.59
GGT (IU/L)	585.6±427.5	500.1±312.2
ALP (IU/L)	650.5±466.9	557.8±358.6
PT-INR	1.047±0.212	1.167±0.477
Platelet count (10^9^/L)	481.3±152.9	293.1±193.8

LSM, liver stiffness measurement; AST, aspartate aminotransferase; ALT, alanine aminotransferase; GGT, gamma glutamyl transpeptidase; ALP, alkaline phosphatase; PT-INR, prothrombin time-international normalized ratio.

### Comparison Between Groups with and without LRE

Among total 69 patients, 27 (39.1%) cases showed LRE development (**Table 2**). At the time of the Kasai operation, the mean age of the patients with LRE development was older than that of the patients without LRE (14.04±8.13 versus 7.85±4.00, P<0.001). AST and ALT were higher (208.0±112.3 versus 107.0±87.00, 183.0±99.9 versus 89.9±71.1, P<0.001) and direct bilirubin was also higher in the group with LRE (8.64±2.78 versus 6.00±2.21). The median LSM values were 21.7 kPa versus 6.7 kPa at baseline, with a higher value in patients who developed LRE during follow-up. Furthermore, the total bilirubin, GGT, and PT-INR results showed a significant difference between the two groups.

**Table 2:** Comparison between patients with and without LRE.

Characteristics	At the time of Kasai			At 3 months after Kasai		
	LRE (N = 27)	Non-LRE (N = 42)	P	LRE (N = 27)	Non-LRE (N = 42)	P
Age (weeks)	14.04±8.13	7.85±4.00	<0.001			
METAVIR fibrosis, 3/4	4/6	4/4	0.004			
LSM value (kPa)	21.7 (4.1–45.0)	6.7 (3.1–45.0)	<0.001	34.8 (13.7–75.0)	13.1 (3.5–22.2)	<0.001
AST (IU/L)	208.0±112.3	107.0±87.0	<0.001	245.4±184.6	127.4±80.4	0.001
ALT (IU/L)	183.0±99.9	89.9±71.1	<0.001	196.2±102.4	144.5±97.7	0.039
Albumin (g/dL)	3.84±0.55	3.78±0.35	0.599	3.03±0.62	3.78±0.61	<0.001
Total bilirubin (mg/dL)	10.44±3.19	8.00±2.48	0.001	9.50±5.34	1.78±1.85	<0.001
Direct bilirubin (mg/dL)	8.64±2.78	6.00±2.21	<0.001	8.12±4.70	1.38±1.55	<0.001
GGT (IU/L)	671.5±425.7	530.3±424.5	0.182	650.2±250.4	403.6±312.2	0.001
ALP (IU/L)	795.3±488.6	557.4±432.9	0.038	657.9±396.2	493.5±320.8	0.063
PT-INR	1.113±0.272	1.005±0.152	0.038	1.431±0.671	0.998±0.126	<0.001
Platelet count (10^9^/L)	499.8±191.4	469.5±123.1	0.425	252.9±198.1	323.8±188.0	0.139

LRE, liver related events; LSM, liver stiffness measurement; AST, aspartate aminotransferase; ALT, alanine aminotransferase; GGT, gamma glutamyl transpeptidase; ALP, alkaline phosphatase; PT-INR, prothrombin time-international normalized ratio.

For the two groups, the differences between before and 3 months after the Kasai operation were similar (**Table 2**). AST and ALT were more elevated in patients with LRE. The total-bilirubin, direct-bilirubin, and GGT were also higher in patients with LRE. Albumin was significantly lower in patients who had developed LRE. The median LSM at 3 months after surgery (34.8 kPa) was higher in patients with LRE than in patients without LRE development (13.1 kPa).

### Patient Characteristics at Follow-Up

At the follow-up assessment, mean age was 5.4±3.7 years, and the duration after the Kasai HPE ranged from 75 weeks to 445 weeks. Among 69 patients, 27 patients developed LRE; 9 cases of jaundice, 4 cases of ascites, 7 cases of variceal bleeding, 9 patients expired due to complications of chronic liver disease, and 2 cases of combined events (**Table 3**). There was no hepatic encephalopathy and hepato-renal syndrome in the patients during follow-up. AST, total-bilirubin, and direct-bilirubin during follow-up were more elevated in patients with LREs than in patients without LRE. Albumin was significantly lower in the LRE group while GGT and PT-INR was significantly higher in the LRE group compared with the non LRE group. LRE developed an average of 32.5±29.4 months after the operation. Eleven children underwent liver transplantation during the follow-up period.

**Table 3:** Follow-up characteristics of the patients.

Characteristics	Total patients (N = 69)	LRE (N = 27)	Non-LRE (N = 42)	P
Age (years)	5.4±3.7	5.3±3.2	5.4±3.9	NS
AST (IU/L)	173.6±142.5	221.9±107.5	124.3±173.8	0.032
ALT (IU/L)	164.7±102.1	177.9±105.8	103.9±147.1	0.059
Albumin (g/dL)	3.45±0.71	3.33±0.50	3.86±0.63	0.003
Total bilirubin (mg/dL)	4.79±5.24	8.91±5.32	1.47±2.64	<0.001
Direct bilirubin (mg/dL)	4.05±4.59	7.29±4.39	1.05±2.21	<0.001
GGT (IU/L)	500.1±312.2	609.6±596.4	240.0±389.6	0.006
ALP (IU/L)	557.8±358.6	609.2±510.5	427.5±429.9	0.162
PT-INR	1.167±0.477	1.643±1.011	1.148±0.443	0.01
Platelet count (10^9^/L)	296.1±193.8	192.3±112.3	232.1±114.7	0.219
Onset duration of LRE (months)		32.5±29.4		
Modes of LRE				
Jaundice	9/69	9/27	0/42	
Ascites	4/69	4/27	0/42	
Variceal bleeding	7/69	7/27	0/42	
Liver related death	9/69	9/27	0/42	

LRE, liver related events; LSM, liver stiffness measurement; AST, aspartate aminotransferase; ALT, alanine aminotransferase; GGT, gamma glutamyl transpeptidase; ALP, alkaline phosphatase; PT-INR, prothrombin time-international normalized ratio.

### Independent Risk Factors of LRE Development

As shown in **Table 3**, LSM value, AST, ALT, albumin, total-bilirubin, direct-bilirubin, and PT-INR significantly differed among patients with and without LRE. The multivariate analysis identified LSM value as the most powerful independent factor for the development of LRE (P = 0.018, HR, 1.258; 95% CI, 1.032–1.522; **Table 4**).

**Table 4:** Univariate and multivariate analysis to identify independent risk factors for LRE development.

Variables	Univariate	Multivariate		
(at 3 months after Kasai)	P	P	Hazard ratio	95% confidence interval
LSM value (kPa)	<0.001	0.018	1.258	1.032–1.522
AST (IU/L)	0.001	0.369	1.006	
ALT (IU/L)	0.039	0.323	0.992	
Albumin (g/dL)	<0.001	0.538	0.529	
Total bilirubin (mg/dL)	<0.001	0.054	1.635	
Direct bilirubin (mg/dL)	<0.001			
ALP (IU/L)	0.063			
Platelet count(10^9^/L)	0.139			

LRE, liver related events; LSM, liver stiffness measurement; AST, aspartate aminotransferase; ALT, alanine aminotransferase; GGT, gamma glutamyl transpeptidase; ALP, alkaline phosphatase.

### Accuracy of LSM Cut-off Value of 3 Months after Kasai Operation in Predicting LRE

**Figure 1:**
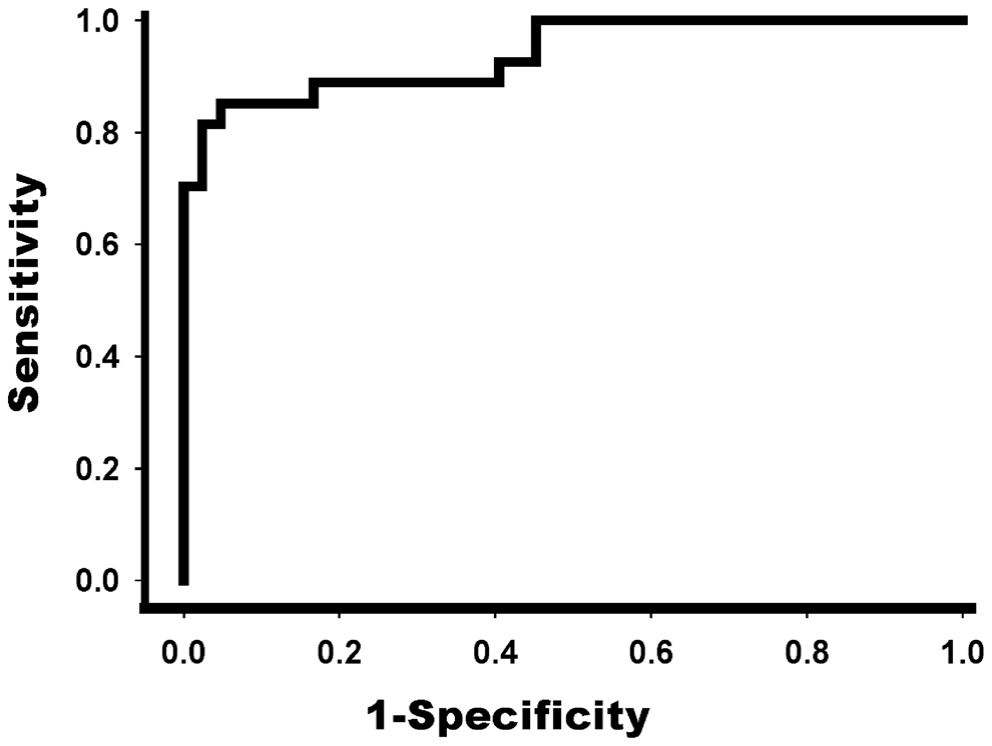
Receiver operation characteristics (ROC) curve for LSM of 3 months after HPE. The area under the ROC curve was 0.943 for the determination of LRE and the optimal cutoff value was 19.9; HPE, hepatoportoenterostomy.

**Figure 2:**
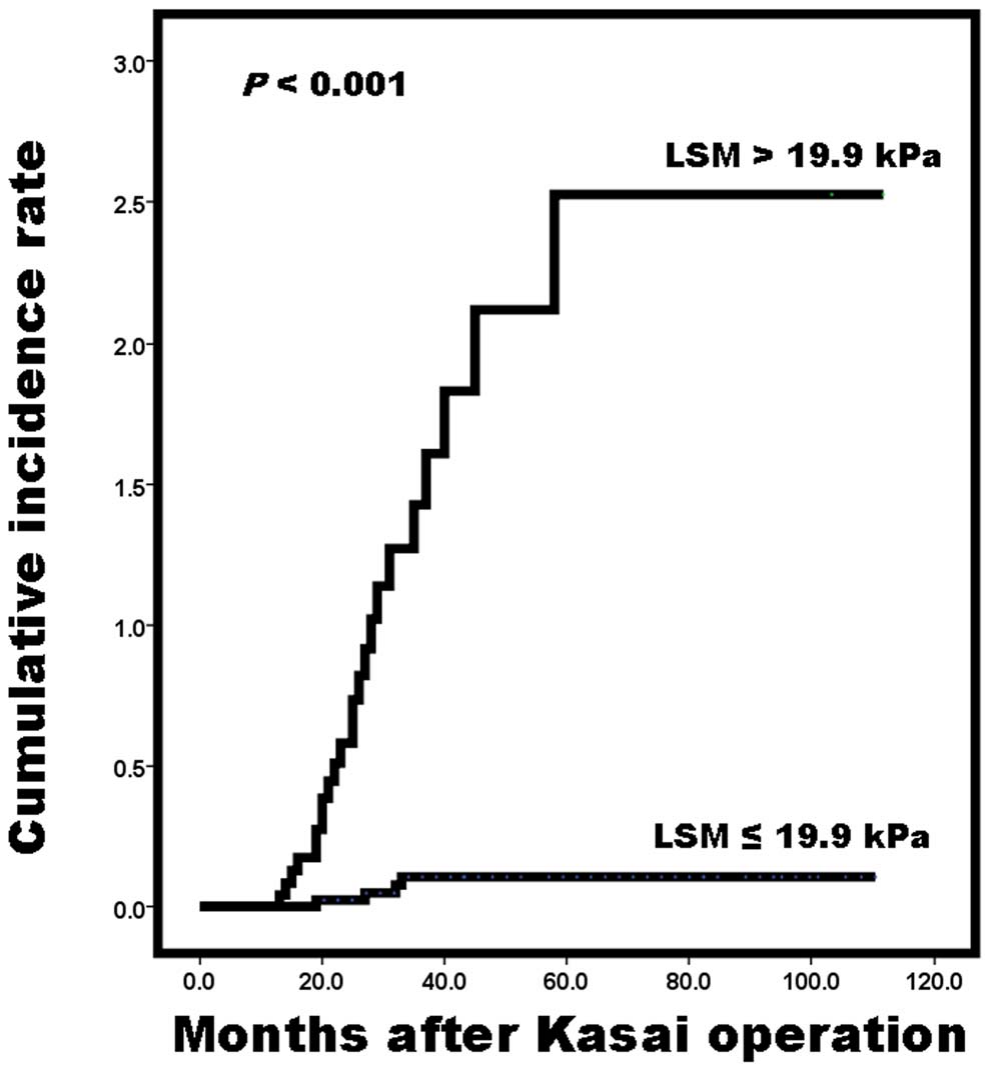
Cumulative incidence rates of LRE based on LSM values. Patients with LSM>19.9 kPa were at greater risk of LREs development compared with those with LSM≤19.9 kPa. LRE, liver related event; LSM, liver stiffness measurement.

**Table 5** shows the optimal LSM cut-off value for all subjects, as well as the corresponding sensitivity and specificity. The clear cut-off value (19.9 kPa) was obtained for LRE development with a total sensitivity and specificity of 1.804. Furthermore, the positive predictive value (PPV) and negative predictive value (NPV) for the 19.9 kPa cut-off value of the LSM were 92.0% and 90.9%, respectively for the prediction of LRE. Using ROC curve analysis to determine the optimal LSM cut-off value for partitioning the two groups, 19.9 kPa showed the most accuracy (**Figure 1**). AUROC resulted in 0.943, with sensitivity of 85.3% and specificity of 95.2%. Twenty-five patients with LSM values <19.9 kPa were showed to have greater risk of LRE development (P<0.001) in comparison with forty-four patients with LSM values ≤19.9 (**Figure 2**).

**Table 5:** Accuracy of LSM cut-off value of 3 month after Kasai operation in predicting LRE.

LSM (kPa)	All patients (N = 69)	LRE (N = 27)	Non-LRE (N = 42)	Sensitivity	Specificity	PPV	NPV
	N (%)	N (%)	N (%)	%	%	%	%
≤19.9	44 (63.8)	4 (14.8)	40 (95.2)	85.2	95.2	92.0	90.9
>19.9	25 (36.2)	23 (85.2)	2 (4.8)				

LRE, liver related events; LSM, liver stiffness measurement; PPV, positive predictive value; NPV, negative predictive value.

## Discussion

Biliary atresia is a disease with a progressive and inflammatory course resulting in irreversible cholestasis and end stage liver disease even with early surgical interventions. Multifactorial pathways lead to immune-mediated intrahepatic and extrahepatic damage of bile ducts, ductular proliferation and finally progressive portal fibrogenesis. [22] The exact pathogenesis of the progressing hepatic fibrosis in biliary atresia is not well known but recent progressions have been made to reveal some key factors and markers associated with fibrogenesis.

*Honsawek et al*. [23] proposed a positive correlation between the serum osteopontin level and the progression of hepatic fibrosis in biliary atresia patients, serum adiponectin, and gelectin-3 as a marker of liver stiffness. [24], [25] These studies use LSM, which has become a reliable and popular tool in assessing hepatic fibrosis, as a comparative factor; however, further clinical validations and applications with LSM are currently being discussed.

Extrahepatic cholestasis is one of the factors that affect LSM, [26] therefore, LSM values with an IQR/M below 0.3 were selected to minimize the influence of cholestasis.

Since the introduction of LSM, it has been shown that LSM can overcome the limitations of liver biopsy and other conventional biomarkers for its noninvasiveness and reproducibility; many reports have been documented that compare LSM with other clinical data of end stage liver disease patients and have proven the usefulness of LSM especially in adults. Transient elastography can be used to monitor disease progression or predict the development of liver-related complications. [24] Over the past five years, reports focusing on the risk assessment of esophageal/gastric variceal bleeding among children with transient elastography in biliary atresia children have been documented. [25], [27] Esophageal/gastric varices are one of the life threatening complications of portal hypertension, and studies have revealed that LSM is a useful tool in predicting esophageal/gastric varices since its result correlates with the presence of varices. In the reports, comparisons of LSM were conducted between groups who have varices and groups who do not have varices; *Chongsrisawat et al*. [15] showed that the liver stiffness score was higher in patients with esophageal and/or gastric varices than in the control group without varices (LSM 37.72±21.55 versus 10.97±8.71 kPa; P<0.001), while *Chang et al*. [16] also reported similar results showing LSM for the group with varices and for without varices of 21.35±10.31 and 9.75±8.61, respectively (P<0.001).

While this study was performed retrospectively, it is unique because we predicted LRE with the LSM values that were collected 3 months after the Kasai operation in each child. Three months is accepted as the duration of hepatic congestion and cholestasis after the surgery that requires resolution, and successful bile flow is an important factor for prognosis in biliary atresia patients. In a previous report, the resolution time of the jaundice after Kasai operation was an average of 60–70 days. [28] Moreover, it was suggested that by 3 months after HPE, a clear difference in total bilirubin levels was evident between children with good outcomes and bad outcomes. [29] We found that patients with high LSM 3 months after the surgery showed a tendency for higher LSM during follow-up, finally resulting in a higher incidence of LRE. Previous studies were focused mainly on esophageal and/or gastric varices among various LRE, while this study showed a clinical implication including other LRE. Patients with not only varices but also other LRE following biliary atresia such as ascites, prolonged jaundice, and death were counted and that LSM values among the two groups (with or without LRE) have been compared to set up the cut off value.

The group with high LSM and abnormal lab results (high AST and ALT, low platelet count, and prolonged PT) before surgery showed similar results 3 months after the surgery and during follow-up. Among the several variables, LSM after 3 months of surgery was the most powerful tool in predicting LRE and prognosis of the patients. Using an ROC curve, the LSM value of 19.9 kPa was suggested as the cut-off value to predict LRE. Moreover, patients with LSM values >19.9 kPa were found to have more risk of LRE development. Through this result we propose more intense preemptive care for patients with high possibility of LRE after Kasai HPE.

Patients with a high LSM value 3 months after surgery finally showed a higher LRE value. Therefore, more attention, frequent follow-up, and monitoring for these patients is recommended.

There are a number of limitations to this study: more patients are needed to represent a more reliable study, the cut-off value of 19.9 kPa is a relatively limited result based on the data of 69 patents, the data from a large number of patients are required to establish a more accurate cutoff value. And the study was performed retrospectively, patients who are not eligible to the study have been excluded at first, this might have resulted in bias. Moreover, follow-up duration after the Kasai HPE was different among each patient.

In conclusion, since LSM after 3 months of surgery correlates with LRE, our study suggests that LSM would provide clinical benefit giving family and care providers advance warning of the anticipated course potentially allowing patients to be treated more aggressively to lessen the complications of end-stage liver disease during follow-up of biliary atresia after their Kasai operation. However, larger scale prospective studies and long term follow-up would be needed to establish a reliable reference for LSM.
